# Variant Interpretation for Cancer (VIC): a computational tool for assessing clinical impacts of somatic variants

**DOI:** 10.1186/s13073-019-0664-4

**Published:** 2019-08-23

**Authors:** Max M. He, Quan Li, Muqing Yan, Hui Cao, Yue Hu, Karen Y. He, Kajia Cao, Marilyn M. Li, Kai Wang

**Affiliations:** 1 Simcere Diagnostics Co., Ltd., Nanjing, 210042 Jiangsu China; 2 State Key Laboratory of Translational Medicine and Innovative Drug Development, Nanjing, 210042 Jiangsu China; 3 Raymond G. Perelman Center for Cellular and Molecular Therapeutics, Children’s Hospital of Philadelphia, Philadelphia, PA 19104 USA; 4 Department of Population and Quantitative Health Sciences, Case Western Reserve University, Cleveland, OH 44106 USA; 5 Division of Genomic Diagnostics, Department of Pathology and Laboratory Medicine, Children’s Hospital of Philadelphia, Philadelphia, PA 19104 USA; 6 Department of Pathology and Laboratory Medicine, Perelman School of Medicine, University of Pennsylvania, Philadelphia, PA 19104 USA

**Keywords:** Somatic variant interpretation, Genetic diagnosis, Cancer genetics, Standards and guidelines

## Abstract

**Background:**

Clinical laboratories implement a variety of measures to classify somatic sequence variants and identify clinically significant variants to facilitate the implementation of precision medicine. To standardize the interpretation process, the Association for Molecular Pathology (AMP), American Society of Clinical Oncology (ASCO), and College of American Pathologists (CAP) published guidelines for the interpretation and reporting of sequence variants in cancer in 2017. These guidelines classify somatic variants using a four-tiered system with ten criteria. Even with the standardized guidelines, assessing clinical impacts of somatic variants remains to be tedious. Additionally, manual implementation of the guidelines may vary among professionals and may lack reproducibility when the supporting evidence is not documented in a consistent manner.

**Results:**

We developed a semi-automated tool called “Variant Interpretation for Cancer” (VIC) to accelerate the interpretation process and minimize individual biases. VIC takes pre-annotated files and automatically classifies sequence variants based on several criteria, with the ability for users to integrate additional evidence to optimize the interpretation on clinical impacts. We evaluated VIC using several publicly available databases and compared with several predictive software programs. We found that VIC is time-efficient and conservative in classifying somatic variants under default settings, especially for variants with strong and/or potential clinical significance. Additionally, we also tested VIC on two cancer-panel sequencing datasets to show its effectiveness in facilitating manual interpretation of somatic variants.

**Conclusions:**

Although VIC cannot replace human reviewers, it will accelerate the interpretation process on somatic variants. VIC can also be customized by clinical laboratories to fit into their analytical pipelines to facilitate the laborious process of somatic variant interpretation. VIC is freely available at https://github.com/HGLab/VIC/.

**Electronic supplementary material:**

The online version of this article (10.1186/s13073-019-0664-4) contains supplementary material, which is available to authorized users.

## Background

With the rapid development of massively parallel next-generation sequencing (NGS) technologies, a large number of cancer genomes, exomes, or gene panels are being sequenced around the world for both biomedical research and clinical diagnosis. DNA sequencing has become an important component in cancer diagnosis and treatment, which facilitates the implementation of precision medicine. However, determining the clinical impacts of somatic variants in cancer presents a different set of challenges from those for germline variants.

Various tools and databases have been developed by different laboratories and institutes, in combination with experts’ opinions, for the interpretation of clinical significance on sequence variants. Annotation tools, such as ANNOVAR [1] and SnpEff [2], as well as many computational prediction algorithms, such as SIFT [3], PolyPhen-2 [4], MutationAssessor [5], MutationTaster [6], and PROVEAN [7, 8], can annotate variants with respect to transcript structure or predicted functional importance; however, they mostly focus on germline variants. Several cancer-specific variant databases have gathered and curated unstructured information on the effectiveness of therapies targeting specific cancer drivers, such as the Catalogue of Somatic Mutations In Cancer (COSMIC) [9], My Cancer Genome (https://www.mycancergenome.org), Clinical Interpretations of Variants in Cancer (CIViC) [10], OncoKB [11], the Precision Medicine Knowledge Base (PMKB) [12], and Cancer Genome Interpreter (CGI) [13]. However, these databases have varying data formats and can often only interpret well-known hotspot somatic variants. Additionally, these databases should be used with caution because they compile information from heterogeneous sources, and many submitted variants lack clinical-grade curation or may only be discovered in exploratory research studies. Therefore, how to comprehensively annotate and interpret the clinical significance of somatic variants is an important yet unresolved challenge.

To standardize the clinical interpretation of cancer genomes, the Association for Molecular Pathology (AMP), American Society of Clinical Oncology (ASCO), and College of American Pathologists (CAP) published standards and guidelines for the interpretation and reporting of sequence variants in cancer in 2017 [14]. The AMP-ASCO-CAP guidelines proposed to categorize somatic variants into a four-tiered categorization system based on their clinical significances, namely strong clinical significance, potential clinical significance, unknown clinical significance, and benign or likely benign. The guidelines also present primary resources for evidence needed to effectively assess the clinical significance of a particular variant. In addition, ClinGen Cancer Somatic Working Group suggested the standards of the interpretation of cancer variants and developed the Minimal Variant Level Data (MVLD) framework to interpret and report clinically actionable drug-associated somatic variants [15, 16].

We have previously developed a variant interpretation framework called InterVar for interpreting clinical significances of germline variants [17] based on the ACMG-AMP 2015 guidelines. InterVar utilizes 28 criteria recommended by ACMG and AMP [18], including 18 automatically generated items and 10 manually adjustable ones. In this study, we used similar procedures to develop a cancer-specific interpretation tool called “Variant Interpretation for Cancer” (VIC) to systematically interpret somatic mutations in cancer. This tool was developed on the basis of the AMP-ASCO-CAP 2017 guidelines [14] and incorporated many resources (e.g., CGI, PMKB, and CIViC) listed on the Global Alliance for Genomics and Health (GA4GH)’s Variant Interpretation of Cancer Consortium (https://cancervariants.org/), but with a strong emphasis on automation of evidence generated from an internal collection of databases and/or a user-defined knowledgebase. It systematically considers seven criteria, including FDA-approved therapies, variant type, population allele frequency, absence/presence in germline and somatic databases, predictive software programs, and pathway involvement to assign scores to each somatic variant and generate the preliminary prediction for clinical impacts. Information suggested by the ClinGen Cancer Somatic Working Group is provided in the final result of VIC, including the allele description, the DNA and protein substitution, the variant type and consequences, and all of the scores of the criteria implemented in the program. This tool assigns somatic variants into four categories: (1) strong clinical significance, (2) potential clinical significance, (3) unknown clinical significance, and (4) benign or likely benign.

## Implementation

### Pre-annotated variants

VIC takes either unannotated VCF files or pre-annotated files generated by ANNOVAR as input files. If a VCF file is not annotated, VIC will automatically call ANNOVAR to generate necessary annotations including refGene, ensGene, knownGene, esp6500siv2_all, 1000g2015aug_all, exac03, gnomad211_exome, avsnp150, dbnsfp35a, dbscsnv11, dbnsfp31a_interpro, clinvar_20190305, cosmic89_coding, icgc21, and so on [1]. We expect that the versions of annotation databases will be updated in a regular basis, given the continuous development of various databases. VIC will then take into account the information as well as some criteria listed below to make the final interpretation. The Java program of VIC is freely available at GitHub (https://github.com/HGLab/VIC/).

### Criteria and scoring system

According to the AMP-ASCO-CAP 2017 guidelines, there are a total of ten types of evidence to predict the clinical significance for somatic variants, including therapies approved by the FDA or reported in professional guidelines, investigational therapies, mutation type, variant allele fraction (mosaic variant frequency (likely somatic), non-mosaic variant frequency (potential germline)), population databases (absence or extremely low minor allele frequency), germline databases, somatic databases, predictive results of different computational algorithms, pathway involvement, and publications [14]. Among these criteria, VIC automatically generates evidence on seven criteria according to the current sources, namely FDA-approved therapies for tumor, mutation type, population database, germline database, somatic database, predictive software, and pathway involvement. The remaining three criteria will require manual adjustments by users (“-s evidence_file” option in the program, an example of customized evidence file is provided as Additional file 1). We described the details below on how to assign a score for each criterion from various sources of annotation information.

### Clinical impacts

The interpretation of somatic variants needs to be focused on their clinical impacts. A variant can be considered as a biomarker for guiding a clinical treatment if it alters the function of a gene or can be targeted by approved or investigational drugs, or predict sensitivity, resistance, and toxicity to a specific drug/therapy. On the basis of the guidelines [14], the clinical significance of a variant is categorized into four levels: (A) biomarkers that predict response or resistance to therapies approved by the FDA (https://www.fda.gov/Drugs/ScienceResearch/ucm572698.htm) or included in the professional guidelines (PG) for specific types of tumors such as the National Comprehensive Cancer Network (NCCN) guideline (https://www.nccn.org/professionals/physician_gls/default.aspx), or act as diagnostic and/or prognostic biomarkers in PG for certain types of tumors; (B) biomarkers based on well-powered studies with experts’ consensus or smaller studies that are repeatedly confirmed or reproduced by different groups; (C) FDA-approved therapies or therapies included in PG for a different tumor type, or investigative therapies with some clinical evidence, or diagnostic and/or prognostic biomarkers with significance based on the results of multiple small studies; (D) biomarkers that have been associated with targeted therapies in preclinical studies with plausible effects, or biomarkers that assist with diagnosing or prognosing diseases themselves or along with other biomarkers on the basis of small studies or some case reports. We complied data from PMKB [12] and CGI [13] into our therapeutic database in the VIC software tool. If the evidence is categorized as level A or B, which is listed as “Tier 1” in the guideline [14], then those variants are considered to have strong clinical significance and are assigned a score of 2 (documented as “guidelines” or “approved” in the CGI for a specific cancer type). However, if a variant is listed as “FDA guidelines” or “approved” in the CGI but the user-defined cancer type of interest is not the corresponding one in the guidelines, then a score of 1 will be assigned. The variants with potential clinical significance falling into level C or D (Tier 2) are given a score of 1 (documented as “preclinical” or “case report” or “trials” in CGI or recorded in the PMKB), while the remaining variants (either with unknown clinical significance or benign/likely benign) are given a score of 0. Meanwhile, the corresponding therapeutic evidence in CGI and clinical evidence summaries from CIViC [10, 15] are incorporated into the final result of VIC if there are any.

### Mutation type

The type of mutation, such as the likely loss-of-function (LoF) variants [19], nonsynonymous SNVs, CNVs, and gene fusions, as well as the major function of the gene (activating or tumor suppressors) are considered, and they can be automatically evaluated in VIC. The required annotation information is generated from ANNOVAR. Currently, 4865 genes were retrieved from the data in ClinVar [20] and ExAC [21], and they were used as our LoF-intolerant genes. Null variants (namely frameshift, splice, stop-gain, and stop-loss variants) in these genes are considered as likely LoF variants. In the VIC scoring procedure, likely activating and LoF mutations are given a score of 1, whereas the variants with functions annotated as unknown, benign, missense, or other types are marked as 0. Note that splice variants are annotated as those that disrupt canonical splice sites within 2 bp of the exon/intron boundaries. The prediction of the impact of exonic missense variants on splicing is generated by the “dbscsnv11” database in ANNOVAR [1]. More details are elucidated in the “Criteria and Scoring System” section in Li et al. [17].

### Variant allele frequency/fraction and potential germline variants

The variant allele frequency (VAF; also known as variant allele fraction) is used to infer whether a variant comes from somatic cells or inherited from parents when a matched normal sample is not provided.

A variant is potentially a germline mutation if the VAF is approximately 50% or 100%. However, certain germline variants, such as large insertions or deletions (INDELs), may cause preferential amplification or capture of normal homologue, resulting in < 50% VAF for germline variants. Laboratories should have clear criteria to differentiate between somatic and germline mutations if a matched normal sample is not available. When a pathogenic germline variant is suspected during tumor-only testing, the variant is recommended to be confirmed with a paired-normal sample according to the AMP-ASCO-CAP guidelines [14]. The laboratories could always set their own criteria, such as adjusting the VAF based on copy number information or mutation type, i.e., SNV or INDEL, to differentiate the somatic variants. Because this element relies highly on the laboratory sequencing protocols and the optimal thresholds may vary greatly between laboratories, this part is not implemented in VIC, but users can adjust the corresponding scores with customized evidence file (Additional file 1) to help differentiate and facilitate the subsequent analysis. Furthermore, users can perform their own data pre-processing step to filter out possible germline variants and only keep predicted somatic mutations, then perform the interpretation in VIC. For the interpretation of germline sequence variants, the ACMG/AMP standards and guidelines [18] should be referred to.

### Population database

The frequencies of minor alleles in control populations are useful for assessing clinical significances of somatic variants. Population databases can be used to filter out variants that are deemed polymorphic/benign based on an arbitrary cutoff of minor allele frequency (MAF). Researchers usually use MAF = 0.01 as the cutoff; however, the optimal threshold can vary. It is recommended to assign different cutoff values to various populations/cohorts. If a variant is absent in a large control cohort or present at extremely low frequencies, there could be a piece of evidence for pathogenicity or clinical impact. VIC uses four databases to assess the MAF, including the 1000 Genomes Project [22], the Exome Aggregation Consortium (ExAC) [21], the NHLBI GO Exome Sequencing Project (ESP6500) [23], and the Genome Aggregation Database (gnomAD 2.1.1) [21]. If a variant is absent in all control subjects or the variant has very low MAF (e.g., < 0.005), it is marked as 1. In comparison, if the MAF in the general population is > 0.01, it is marked as 0.

### Germline mutation database

Germline mutation databases, such as ClinVar [20], are useful resources for evaluating variants in genes associated with cancer predisposition syndromes or well-studied germline counterparts. VIC takes the annotation from CLINSIG as one of the references (annotation of clinical significance in ClinVar, https://www.ncbi.nlm.nih.gov/clinvar/docs/clinsig/), assigns a score of 2 for variants with only pathogenic and no benign evidence, a score of 1 for benign or likely benign observations, and a score of 0 for either contradictory cases (both benign and pathogenic reported) or uncertain/unknown significance. The score of this parameter will be compiled with those of other criteria to make the final interpretation.

### Somatic mutation database

Somatic mutation databases, such as the COSMIC [9], My Cancer Genome (https://www.mycancergenome.org), the International Cancer Genome Consortium (ICGC) [24], and The Cancer Genome Atlas (TCGA) [25], contain most somatic variants observed/reported by researchers or clinical professionals, especially those that are highly associated with disease, diagnosis, or therapies. Currently, VIC adopts COSMIC (v89) and ICGC as somatic databases to interpret the clinical significances of somatic variants. We assign a score of 2 for variants present in both databases, 1 for variants present in only one database, and 0 for variants absence in both databases.

### Predictive software programs

For missense variants, a variety of tools can predict the likelihood of whether a given variant is damaging to protein function or structure by using evolutionary information, context within the protein sequence, and biochemical properties based on the probabilistic assertions. Most tools are designed for optimal performance on germline variants rather than somatic variants, and the impacts on protein function do not necessarily translate to pathogenicity on human diseases. These computational methods include individual scoring algorithms or systems, such as SIFT [3], PolyPhen-2 [4], MutationAssessor [5], MutationTaster [6], and FATHMM [26], as well as meta-predictors like MetaSVM [27]. Because of their individual limitations, e.g., some are biased toward protein structures and evolutionary information, it is recommended that the results of these prediction algorithms should never be used as the sole evidence for variant classification or clinical decision-making. By default, VIC integrates the prediction of seven tools, including MetaSVM, SIFT, Polyphen-2, MetaLR [27], FATHMM, MutationTaster, and GERP++ [28], and a score of 2 is given if more than three tools suggest that a given variant is damaging. A score of 1 is assigned when the variant is predicted as damaging or benign by an equal number of tools. If a variant is marked as benign/likely benign by more than three algorithms, then it is assigned a score of 0. The scoring logic can be adjusted by users to fit their specific needs.

### Pathway involvement

Nonsynonymous mutations in key genes involved in biological pathways will adversely affect the metabolism, signal transduction, or cellular function and consequently may contribute to cancer initiation and progression. Understanding the functions of major genes in a pathway is critical in locating targeted drug-associated mutations. Two internal gene lists were built from the Cancer Gene Census (CGC, https://cancer.sanger.ac.uk/census) [29] and the Kyoto Encyclopedia of Genes and Genomes (KEGG, https://www.genome.jp/kegg-bin/show_pathway?hsa05200) to measure the involvement of genes in cancer-related pathways. In total, 576 CGC Tier 1 genes classified based on COSMIC v89 were selected to build the “cancer_genes.list” file as one of VIC’s internal databases; these genes have documented cancer-related activities and evidence of mutations in promoting oncogenic transformation. Meanwhile, 1581 genes involved in cancer pathways were retrieved from KEGG, and we organized these genes into the file “cancer_pathways.list.” If a given mutation is located in a critical gene in a cancer-associated pathway (genes in cancers_genes.list), it is marked with a score of 2; if a variant is present in a gene potentially involved in a cancer-associated pathway (genes in cancer_pathways.list), it is marked with a score of 1; as for variants in genes with unclear functions, they are marked with a score of 0. We acknowledge that the gene list and pathway list may not be comprehensive/accurate and may not be optimal when specific types of cancer are examined, and users can substitute their own gene/pathway list in the VIC software.

### Publications

Researchers and clinical professionals publish their research and clinical trial results to share their discoveries with the scientific community. One could study the function and clinical impact of a variant or gene by searching scientific publications. However, the conclusions may vary among studies due to different study designs, methods, populations, and objectives. Because it requires a massive workload to search and filter out the relevant publications, VIC currently does not automatically score a variant based on this criterion. Nevertheless, VIC presents available publications documented in CIViC in the final results (to facilitate human reviewers make judgment); moreover, users can compile a private collection of variants of interest and VIC will take the clinical interpretation defined by users in addition to providing other annotations in the final result. This function can be activated by the “-l user_specified_list” option, and an example of the “known-list file” is provided as Additional file 2.

### Scoring system

VIC implemented its scoring system based on the seven criteria. Among them, the therapies, mutation type, population data, somatic data, and pathway need to meet the basic criteria while the germline data and predictive software can be optional. The basic scoring system is described in more details in Table 1 and Additional file 3. As most of the predictive software programs only work well for SNVs rather than INDELs, VIC currently classifies clinical significance better for SNVs than for INDELs based on available databases and resources. Given user-provided INDEL information (−l option in the program, Additional file 2), VIC can potentially perform better classification on INDELs.

**Table 1:** The scoring system of VIC’s automated step

Evidence (scores)	Strong clinical significance	Potential clinical significance	Benign or likely benign	Uncertain significance
Therapeutic (2/1/0)	2	2/1	0	All other situations
Mutation type (1/0)	1	1	0	
Population data (1/0)	1	1	0	
Somatic data (2/1/0)	2	2/1	0	
Pathway (2/1/0)	2	2/1	0	
Germline data (2/1/0)	2	> 1*	1/0	
Predictive software (2/1/0)	2		1/0	

*The sum of the scores of germline database and predictive software programs

### VIC software implementation

VIC is a command-line-driven software program implemented in Java and can be used as a standalone application on a variety of operating systems that support Java. The pre-annotated files generated by ANNOVAR or unannotated files in VCF format or ANNOVAR input format (avinput) can be taken as input files. If the input files are unannotated, VIC will automatically call ANNOVAR to generate the pre-annotated files. Next, VIC takes the seven criteria as the default setting and performs assessment based on internal resources. After the step-one assessment, users could manually adjust each criterion for re-interpretation. In the output file, each variant will be assigned as one of the four tiers following the AMP-ASCO-CAP 2017 guidelines [14].

## Results

### Summary of the interpretation procedure

**Fig. 1:**
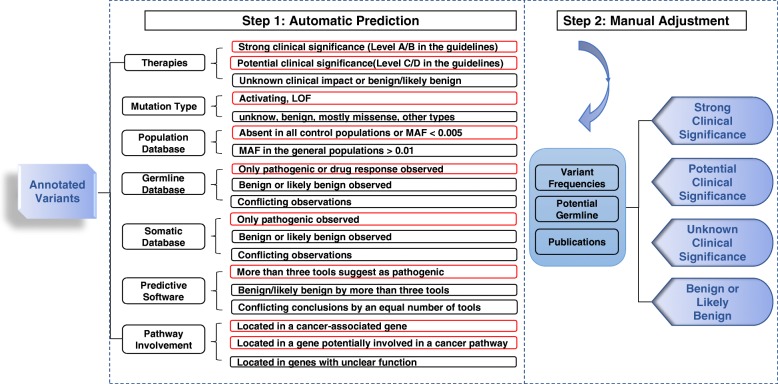
Flowchart of the two-step procedure of VIC

The analytical procedures and scoring logic of VIC are shown in Fig. 1. VIC follows a two-step procedure: (1) generation of scores based on seven criteria and (2) manual adjustment on individual criterion to reach the final conclusion. In the first step, VIC gathers the required annotation information from external software programs and internal resources to generate required evidence scores on seven criteria (see the “Implementation” section). VIC performs a preliminary interpretation of the variants based on all of the information available. Based on additional domain knowledge and patient-specific information, users can perform manual adjustment in the second step, modify existing scores on existing criteria, and assign new scores on additional criteria. Finally, VIC then takes scores of each criterion and assigns clinical significance for somatic variants based on the AMP-ASCO-CAP 2017 guidelines.

We herein illustrated the procedure for the assessment of one variant in a step-by-step fashion. This is a missense variant located in exon 21 of *EGFR* (GRCh37, chr7:55259515–55259515T> G, MIM: 131550) [30], which is known to be associated with non-small cell lung cancer (NSCLC). The scoring logic for this variant is as follows: (1) It is a nonsynonymous and known activating mutation, so mutation type score = 1. (2) It is approved by FDA as a biomarker for targeted therapy and is responsive to the tyrosine kinase inhibitor (TKI) (https://www.lls.org/leukemia/chronic-myeloid-leukemia/treatment/tyrosine-kinase-inhibitor-tki-therapy), so therapeutic score = 2. (3) It activates the downstream pathway of *EGFR* and leads to carcinogenesis, so pathway score = 2. (4) It is absent in the 1000G, ExAC, ESP6500, or gnomAD databases; thus, it is likely a rare variant (in the general population), population database score = 2. (5) It is recorded in the COSMIC, ICGC, and CLINSIG as pathogenic, so somatic databases score = 2. (6) Multiple bioinformatics tools (e.g., FATHMM, CADD, SIFT, and PolyPhen2) predict this variant as deleterious, predictive software score = 2. Finally, after manual review, no scores are changed or added, and this variant is classified as strong clinical significance by VIC. Therefore, VIC speeds up variant interpretation by the compilation of information from various sources and the final assignment of four tiers from 10 criteria. In a typical modern computer, it takes about 20 min for VIC to completely interpret 215,000 variants followed by manual review. In comparison, it takes ~2 h for an experienced clinical geneticist to interpret a report on a gene panel containing about 100 variants.

### Comparative analysis with the FATHMM-MKL interpretation in COSMIC

The COSMIC database [9] is the largest and likely the most comprehensive resource for exploring the clinical impacts of somatic mutations in various types of cancer. It includes the predictive results of the FATHMM algorithm for the interpretation of somatic mutations. Variants with FATHMM scores greater than 0.5 are classified as “deleterious”, those with scores greater than or equal to 0.7 are considered “pathogenic”, and those with scores less than or equal to 0.5 are classified as “neutral”. COSMIC provides many different types of data files, and we used the CosmicMutantExportCensus dataset and extracted information from CosmicCodingMuts.vcf (https://cancer.sanger.ac.uk/cosmic/#, version 89, last accessed in June 2019). In total, we retrieved 272,560 unique variants from COSMIC v89 categorized in the following three categories: (1) “pathogenic”, (2) “neutral”, and (3) “none” (no annotation information) by COSMIC. Then, we analyzed these variants using VIC independently.

For the “pathogenic” category (173,150 variants) in the COSMIC, VIC (automated step) classified 6/14, 783 (14,789, 8.54%) variants as strong/potential clinical significance, and the rest as uncertain significance (158,353, 91.45%) or benign/likely benign (8, 0.0046%). In the “neutral” category of COSMIC (50,475 variants), VIC (automated step) classified 416 (0.82%) variants as potential clinical significance, 50,026 (99.11%) variants as uncertain significance, and the rest 33 variants as benign/likely benign (0.065%). In addition, for the 48,935 variants without any classification labels in the COSMIC database, VIC (automated step) classified 567 (1.16%) as potential clinical significance and 5 as benign/likely benign (0.01%) and classified the remaining variants (48,363, 98.83%) as uncertain significance. In summary, among 272,560 coding variants retrieved from COSMIC, VIC identified 15,772 variants as having strong or potential clinical significance, 256,742 as uncertain significances, and 46 as benign/likely benign (Table 2). These classifications suggest that VIC (automated step) gives far more conservative classifications than COSMIC.

**Table 2:** Summary of variant classification in COSMIC and VIC

VIC	COSMIC			Total
	Pathogenic	Neutral	None*	
Strong significance	6	0	0	6 (0.00%)
Potential significance	14,783	416	567	15,766 (5.78%)
Uncertain significance	158,353	50,026	48,363	256,742 (94.20%)
Benign/likely benign	8	33	5	46 (0.02%)
Total	173,150 (63.53%)	50,475 (18.52%)	48,935 (17.95%)	272,560

*COSMIC variants without “FATHMM_prediction” records in the database

The disagreement between COSMIC and VIC is not surprising due to the lack of confirmed information of many variants. For instance, the mutation c.962C>T located in the coding region of gene *PABPC1* was recorded as “pathogenic” in COSMIC, but was classified as “uncertain significance” in VIC. Many factors can contribute to this contradictory scenario. The predictive bioinformatics tools in VIC indicated that this variant may alter protein function and the MAF was less than 0.005. However, the biological function of this variant in the cancer-related signaling pathway requires more thorough research. Additionally, there was no available targeted therapy/drugs described for this variant in the FDA or other professional guidelines. Therefore, VIC conservatively categorized this variant as “uncertain significance” due to the lack of strong supporting evidence. On the contrary, variant rs28934571 (GRCh37:17:7577534:C>A) was recorded as “neutral” in COSMIC but rated as “potential clinical significance” by VIC. The differences can be attributed to several reasons: this variant was included in our internal therapeutic database, it was a nonsynonymous SNV in the exonic region of *TP53*, it was absent in the population databases (extremely low MAF), it was present in ICGC, it was predicted as damaging by seven tools, *TP53* was involved in a critical cancer-associated pathway, and there was a “pathogenic” record of this variant in ClinVar. These examples illustrated that VIC uses multiple sources of information to derive a set of evidence to help manual review and interpretation of somatic mutations and ensures reproducibility by documenting the source of the evidence used. It serves a different purpose than typical bioinformatics tools that give in silico prediction on whether a variant is likely to alter protein function.

### Comparative analysis with CGI

The CGI [13] is a platform that systematizes the interpretation of cancer genomes. There are four categories of variants in CGI: known, predicted driver, predicted passenger, and not protein affecting. We downloaded four clinical example datasets as well as the validated oncogenic mutations from the CGI website (https://www.cancergenomeinterpreter.org, last accessed in June 2019) for reclassification of variants with VIC. The four example datasets include annotations of variants in two sets of colorectal cancers, chronic lymphocytic leukemia, NSCLC, and bladder carcinoma. Their catalogues of validated oncogenic mutations aggregate data in the DoCM [31], ClinVar, and OncoKB as well as the results of various publications.

Among all of the 474 variants in the four examples, VIC (automated step) identified 2 variants with strong clinical significance and 6 variants with potential significance, and all of them were also annotated as known or predicted drivers by CGI. Moreover, VIC (automated step) identified no benign/likely benign variant and 465 variants of uncertain significance, whereas CGI marked none of them as known, 8 as predicted driver, and the rest as not protein-affecting or predicted passenger (Table 3). One of the “predicted driver” variants is a nonsynonymous SNV (rs373365649: G>A) in the exonic region of *PTPRU*; due to the lack of supportive evidence from the current germline, somatic, and pathway databases, it was categorized into the uncertain group by VIC. As for the validated oncogenic mutations (a total of 5601 mutations) in CGI, we collected 2543 SNVs out of 2748 somatic variants in the dataset and used VIC to classify their clinical significances. Four variants were categorized by VIC as strong clinical significance and 1121 as potential clinical significances, which is about 44.24% (1125/2543) of the total oncogenic SNV alterations in the CGI’s catalogue, and only one was identified as benign/likely benign by VIC (Table 4). Overall, the results indicated that VIC highly concurs with CGI in the clinical significance group and is generally more stringent than CGI.

**Table 3:** Comparison of classification results between VIC and CGI on four example datasets

VIC	CGI				Total
	Known	Not protein-affecting	Predicted driver (Tier1/2)	Predicted passenger	
Strong clinical significance	2	0	0	0	2 (0.42%)
Potential clinical significance	6	0	1	0	7 (1.48%)
Benign/likely benign	0	0	0	0	0 (0.00%)
Uncertain significance	0	110	8	347	465 (98.1%)
Total	8	110	9	347	474

**Table 4:** Comparisons of classification results between VIC and CGI on validated oncogenic mutations

VIC	Strong clinical significance	Potential clinical significance	Benign/likely benign	Uncertain significance	Total
Validated in CGI	4	1121	1	1417	2543

### Comparative analysis with OncoKB

OncoKB takes into account the information of biological, clinical, and therapeutic resources, FDA labels, NCCN guidelines, selected experts’ recommendations, and the medical literature to provide a four-level evidence classification system to interpret the genomic alterations. We obtained the mutation information of the MSK_IMPACT Clinical Sequencing Cohort project [32] as well as the clinical impact interpretation by OncoKB from the cBioPortal platform (http://www.cbioportal.org/). Datasets of 10 Melanoma and 45 NSCLC cases were downloaded from cBioPortal (http://www.cbioportal.org/study?id=msk_impact_2017, last accessed in June 2019) for this study. For the melanoma cases (285 variants), we identified 13 variants with strong/potential clinical significances whereas OncoKB annotated 9 of them as oncogenic/likely/predicted oncogenic. For the NSCLC project, VIC interpreted 40 of the 244 mutations as strong/potential clinical significances while OncoKB classifies 38 of them as oncogenic/likely oncogenic/predicted oncogenic (Table 5). There was only one “likely neutral” variant (GRCh37, chr4:153249393-153249393 G>T) predicted as potential clinical significant by VIC, because it was present in the therapeutic (PMKB) and somatic database (pathogenic in COSMIC), and the affected gene *FBXW7* was involved in cancer-related pathway, and five computational tools predicted it as deleterious. This analysis demonstrated that VIC (automated step) is slightly more conservative in calling variants as having clinical significance than OncoKB; however, manual adjustment based on prior knowledge may change such classifications to a different tier.

**Table 5:** Comparison of classification results between VIC and OncoKB on two case series

VIC	Melanoma in OncoKB					Total
	Oncogenic	Likely oncogenic	Predicted oncogenic	Likely neutral	Unknown/NA	
Strong clinical significance	1	0	0	0	0	1 (0.35%)
Potential clinical significance	5	3	0	0	4	12 (4.26%)
Benign/likely benign	0	0	0	0	0	0 (0.00%)
Uncertain significance	3	30	5	1	233	272 (95.44%)
Total	9	33	5	1	237	285
VIC	NSCLC in OncoKB					Total
	Oncogenic	Likely oncogenic	Predicted oncogenic	Likely neutral	Unknown/NA	
Strong clinical significance	2	0	0	0	0	2 (0.82%)
Potential clinical significance	15	20	1	1	1	38 (15.57%)
Benign/likely benign	0	0	0	0	0	0 (0.00%)
Uncertain significance	5	39	7	1	152	204 (83.61%)
Total	22	59	8	2	153	244

### Comparative analysis with CIViC

CIViC is a crowd-sourced and expert-moderated public resource for somatic variants in cancer [10]. It adopts five evidence levels to differentiate reported mutations, namely A: validated, B: clinical, C: case study, D: preclinical, and E: inferential. In total, 645 unique SNVs/INDELs from 105 unique genes were retrieved from CIViC website (https://civicdb.org/releases, last accessed in June 2019) and assessed by the VIC program. Almost half of the variants retrieved from CIViC were leveled as “C” (350 as case study), among which 5 had strong clinical significance, 174 had potential clinical significance, and 171 had unknown significance based on the results from VIC (automated step). Among the unique SNVs/INDELs, only 4 were predicted as “benign/likely benign,” whereas 13, 291, and 337 variants were identified as “strong”, “potential”, and “uncertain”, respectively, by VIC (Table 6). Among those benign/likely benign variants (all were listed as level B: clinical in CIViC), two were annotated as intronic mutations, one as ncRNA_exonic (non-coding RNA), and one as synonymous SNV. Another inconsistent example is a splicing variant in gene *DPYD* (rs3918290, chr1:97915614-97915614 C>T (GRCh37)). It was listed as level A in the CIViC but predicted as uncertain by VIC, though it is a rare allele in population databases (MAF < 0.005). The reasons are as follows: this variant was absent in our therapeutic and somatic databases, it was interpreted as pathogenic or benign by an equal number of predictive tools, and it was not documented in the genes involved in cancer-related pathways.

**Table 6:** Comparison of classification results between VIC and CIViC on 645 variants from CIViC

VIC	CIViC*					Total
	A	B	C	D	E	
Strong clinical significance	0	4	5	4	0	13 (2.02%)
Potential clinical significance	1	33	174	81	2	291 (45.12%)
Benign/likely benign	0	4	0	0	0	4 (0.62%)
Uncertain significance	3	51	171	67	45	337 (52.25%)
Total	4	92	350	152	47	645

*Five evidence levels listed in the “ClinicalEvidenceSummaries.tsv” of CIViC

### Evaluation of VIC on a real cancer-panel sequencing dataset

To assess the utility of VIC in analyzing real clinical diagnostic dataset, we analyzed the variants of 100 patients with lung cancer using VIC and compared our results with their clinical diagnostic reports. The sequencing panel includes 19 genes associated with targeted therapies on lung cancer and the clinical reports were provided by a diagnostic laboratory in a double-blinded fashion for comparison with VIC. There were a total of 70 SNVs and 26 INDELs reported as clinically significant in the original laboratory reports, among which 69 SNVs and 19 INDELs were classified as strong/potential clinical significance by VIC, demonstrating a 91.67% concordance between VIC and clinical interpretation by experienced molecular pathologists. Our results also showed a much higher concordance rate for SNVs (98.6% consistent) compared to INDELs (73.1%). This is likely due to some INDELs not being fully and/or correctly recorded by databases/resources that VIC has adopted. For instance, a variant in ERBB2 (c.2310_2311ins GCATACGTGATG, p.E770delinsEAYVM) was classified as “uncertain significance” by VIC because there was no relevant information on therapies for this variant in the VIC internal databases. In addition, several bioinformatics tools predicted this variant as benign or uncertain. Similarly, the only contradicting SNV (ERBB2, p.R678Q), which is classified as uncertain by VIC but clinically significant by the diagnostic report, lacked the therapeutic information in our databases and consequently was categorized as uncertain. However, by customizing the score of therapy (CBP0 in the Additional file 1) to 1 by re-running the VIC with “-s” option, this variant was turned to “potential significance”. This example demonstrated the importance of manual adjustment in reaching a final clinical interpretation on somatic mutations.

In addition, VIC also identified more than 10 variants as strong/potential significant out of 27,078 original variants from all 100 samples, but were not covered in the diagnostic report for somatic mutations. The possible reasons could be that they were not covered in the respective hotspot database at the time of diagnosis or they were not in the targeted gene list associated with targeted therapies on lung cancer or without convincing variant allele frequency/fraction information indicating their somatic origin. There was a potentially significant variant c.261delC in gene *TP53*; however, because this gene was not listed as associated with targeted therapies on lung cancer in the NGS panel, it was not reported. This implies that additional information needs to be collected for enriching VIC’s internal databases and prior knowledge from users. Furthermore, this type of discrepancies emphasizes the importance of integrating experiences from variant assessment experts and the importance of utilizing internal institutional databases. Among the 27 strong and 61 potential significance variants classified by VIC, 85 were classified as “pathogenic” by ClinVar, while 67 were predicted as “damaging” by SIFT, 60 were predicted as “damaging” by PolyPhen-2, and 64 were classified as “pathogenic” in COSMIC (Table 7). In addition, 3 “pathogenic” variants in ClinVar, 1 “damaging” variant predicted by PolyPhen-2, and 1 “pathogenic” variant in COSMIC were classified as uncertain significance by VIC (Table 7).

**Table 7:** Assessment of the lung cancer dataset (100 patients) by VIC, ClinVar, SIFT, PolyPhen-2, and COSMIC. The number of reported variants with strong or potential clinical significance from the diagnostic lab is also listed

	VIC				
	Strong	Potential	Uncertain	Benign/likely benign	Total
ClinVar					
Pathogenic	27	58	3	0	88
Conflict	0	0	0	0	0
Benign	0	0	0	0	0
Uncertain	0	1	0	0	1
Other	0	1	0	0	1
N/A	0	1	5	0	6
SIFT					
Damaging	27	40	0	0	67
Benign	0	1	1	0	2
N/A	0	20	7	0	27
PolyPhen-2					
Damaging	27	33	1	0	61
Benign	0	8	0	0	8
N/A	0	20	7	0	27
COSMIC					
Pathogenic	27	37	1	0	65
Neutral	0	0	0	0	0
None	0	24	5	0	29
N/A	0	0	2	0	2
VIC					
Total	27	61	8	0	96

*N/A* no information/not archived, *None* COSMIC variants without “FATHMM_prediction” records in the database

### Evaluation of VIC on an additional panel sequencing dataset on pediatric cancer

**Fig. 2:**
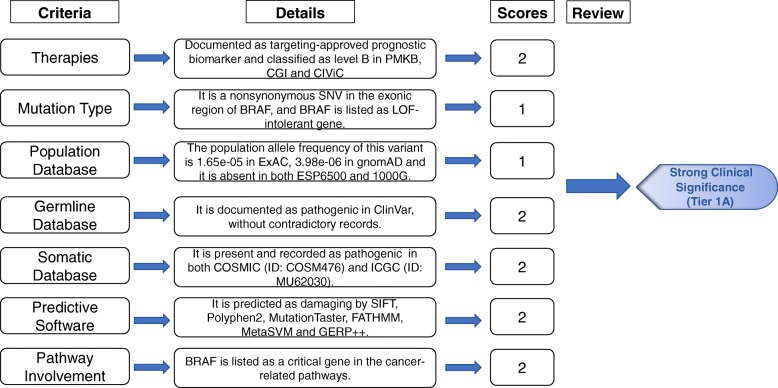
An example illustrating the logic of VIC in the interpretation of a somatic variant in BRAF from an in-house cancer panel sequencing dataset

We further evaluated VIC on five pediatric cancer samples reported as positive at the Children’s Hospital of Philadelphia (CHOP). Among all the 251 somatic variants in the five CHOP samples from panel sequencing data, VIC (automated step) identified 6 variants as strong/potential clinically actionable and 245 as uncertain (Table 8). All six variants with strong/potential clinical impact by VIC were also documented as pathogenic in the COSMIC. The comparisons among VIC, SIFT, PolyPhen, ClinVar, and COSMIC further supports the previous findings that they correlate to each other yet VIC is generally more conservative than other prediction tools. A flowchart illustrating the scoring logic for one of the variants is shown as Fig. 2. We next assessed the original clinical reports on the five patients compiled by experienced clinical geneticists: two patients each had one variant interpreted as tier 1 (strong clinical significance), and all patients have 0, 2, 1, 1, and 2 variants interpreted as tier 2 (potential clinical significance) in the original clinical reports at CHOP. Among them, one INDEL was not interpreted as strong or potential clinical significance by VIC (automated step) due to the lack of documented information on this INDEL, suggesting that VIC may be more conservative on INDELs than SNVs and that it is especially important to examine INDELs in the manual adjustment step in VIC.

**Table 8:** Assessment of the pediatric cancer dataset (5 patients) by VIC, ClinVar, SIFT, PolyPhen-2, and COSMIC. The number of reported variants with strong or potential clinical significance from the diagnostic lab (“REPORT” row) is also listed

	VIC				
	Strong	Potential	Uncertain	Benign/likely benign	Total
ClinVar					
Pathogenic	1	1	16	0	18
Conflict	0	0	4	0	4
Benign	0	0	32	0	32
Uncertain	0	2	11	0	13
Other	0	0	4	0	4
N/A	0	2	178	0	180
SIFT					
Damaging	1	0	73	0	74
Benign	0	5	65	0	70
N/A	0	0	107	0	107
PolyPhen-2					
Damaging	1	5	88	0	94
Benign	0	0	51	0	51
N/A	0	0	106	0	106
COSMIC					
Pathogenic	1	5	42	0	48
Neutral	0	0	26	0	26
None	0	0	15	0	15
N/A	0	0	162	0	162
REPORT					
Strong clinical significance	2				
Potential clinical significance		6			
VIC					
Total	1	5	245	0	251

*N/A* no information/not archived, *None* COSMIC variants without “FATHMM_prediction” records in the database

## Discussion

In the current study, we describe a strategy to implement the ASCO-AMP-CAP 2017 guidelines and present a computational tool to assess the clinical impacts of somatic variants in a semi-automatic fashion. Similar to the InterVar tool that we have previously developed, the goal of VIC is not to replace human acumen in clinical interpretation, but rather to facilitate automatic generation of evidence codes and provide a final summary of the results using evidence codes. We caution that VIC is not designed as a robot with artificial intelligence that gives clinical interpretation automatically, but as a tool to facilitate human beings make clinical judgements. Our comparative analysis with several clinical databases demonstrated that VIC can be utilized in clinical settings to facilitate the somatic variant interpretation process.

We applied VIC to annotate and interpret the variants in COSMIC, CGI, OncoKB, and CIViC, and observed that a small proportion of the variants were classified as strong/potential clinical significances by both VIC and one of the public data resources. This may be due to several reasons. For example, public databases such as COSMIC contain the clinical significance of variants provided by various submitters and some may be simply retrieved from literature without careful curation. Sometimes different scientists use different criteria for assessing the clinical impacts, which leads to different results for a variant. Unsurprisingly, a proportion of variants in these databases might be inaccurately marked as “pathogenic” and are in fact not pathogenic or not related to cancer progression and treatment. On the other hand, it is also possible that some variants classified as tier 3/4 by VIC could be changed to tier 2 (potential clinical significance) if additional evidence-based information is provided by human input (see Additional files 1 and 2 for examples). It is also noticeable that the majority of variants in these databases were classified into tier 3 (unknown clinical significance) by VIC’s automated step, which is probably related to the computational principles of VIC of being conservative in the automated step. VIC designates scores to a variant based on several criteria/resources and cannot give a solid conclusion without strong support from the available resources.

We acknowledge several limitations in the VIC tool. First, VIC provides the level of evidence on the basis of internal databases on the therapeutic context and effect, biomarker class, and the sub-level of evidence recommended by the ClinGen Cancer Somatic Working Group [15, 16]. However, due to the limited resources, some databases that we currently compiled are not comprehensive and may not cover all of the important cancer subtypes; therefore, it may require further manual adjustment on the interpretation and we may include additional high-quality databases in the VIC tool in the future. For example, we employed a therapeutic database compiled from PMKB and CGI, but we were unable to incorporate all databases containing the biomarkers linked with FDA-approved, professional-guided, and investigational therapies. As a result, the internal resources are biased toward the documented variants in our databases. Second, VIC relies on the annotation from ANNOVAR and is only able to manage seven criteria listed in the AMP-ASCO-CAP 2017 guidelines, which is insufficient for many variants and unable to deal with gene fusions and other types of structural variants (SVs); therefore, we design VIC to be flexible on all parameters in order for users to implement their own additional criteria or scoring logic. Furthermore, the AMP-ASCO-CAP guidelines mention ten evidence sources/types for clinical significance interpretation without a specific weight for each criterion, which makes it difficult for the software program to quantify the evidence or weigh different types of evidence differently. In addition, the guidelines do not provide specific rating systems for many criteria, which also hinder the automation process. Therefore, the results generated by VIC should always be used with caution and human review based on professional expertise is required to reach an accurate interpretation; furthermore, an internal collection of variants of interest (such as all reported “positive” variants from a diagnostic lab) is recommended to be used with VIC to take into account of prior knowledge. Additionally, the current VIC software is a command-line-driven tool and may not appeal to users who prefer a graphical user interface. We plan to develop web servers that implement core functionalities of VIC to enable automated annotation of user-supplied VCF files. Finally, the guidelines may evolve in the future, and they may need to integrate larger knowledgebases so that machine learning can replace at least part of rule-making in the current guidelines. Rule-making by itself is a manual process that may not be optimal, but it is a necessary procedure when the amount of training data is not large enough. In summary, based on the AMP-ASCO-CAP somatic variant interpretation guidelines, the two steps in VIC can efficiently assess the clinical impacts of somatic variants and provide users with useful information for further manual interpretation, which significantly increase the efficiency of somatic variant interpretation.

## Conclusions

In summary, we developed VIC to facilitate the assessment of clinical impacts of somatic variants. The VIC tool is built on the AMP-ASCO-CAP 2017 guidelines and generates the preliminary prediction based on seven criteria by default. It also allows users to adjust the parameters manually as an additional step to increase the accuracy of variant interpretation. Although VIC cannot replace human reviewers, it will accelerate the interpretation process on somatic variants. VIC can also be customized by clinical laboratories to fit into their analytical pipelines to facilitate the laborious process of somatic variant interpretation.

### Availability and requirements

Project name: VIC

Project home page: https://github.com/HGLab/VIC/

Operating system(s): Platform independent

Programming language: Java

Other requirements: Java 1.8 or higher

License: GNU GPL

Any restrictions to use by non-academics: license needed

## Additional files

Additional file 1: An example of customized evidence file. (TXT 1 kb)

Additional file 2: An example of the “known-list file”. (TXT 283 bytes)

Additional file 3: More details of the scoring system in VIC. (DOCX 14 kb)

## Data Availability

Due to potential compromise of individual privacy, full datasets of the 100 lung cancer panel from Simcere Diagnostics and the five samples with pediatric cancer panel from the Children’s Hospital of Philadelphia generated and analyzed are not publicly available but are available from the authors on reasonable request and institutional data use agreement. The Java program of VIC is freely available at GitHub (https://github.com/HGLab/VIC/), which includes built-in datasets for variant interpretation. The somatic variants used in the comparative analysis can be obtained in the URLs below, with the version number and accession time documented in the manuscript. CGI: https://www.cancergenomeinterpreter.org/home PMKB: https://pmkb.weill.cornell.edu/ ClinVar: https://www.ncbi.nlm.nih.gov/clinvar/ ExAC: http://exac.broadinstitute.org/ gnomAD: http://gnomad-old.broadinstitute.org/ COSMIC: https://cancer.sanger.ac.uk/cosmic ICGC: https://icgc.org/ CGC: https://cancer.sanger.ac.uk/census KEGG: https://www.genome.jp/kegg/ CIViC: https://civicdb.org/home cBioPortal: http://www.cbioportal.org/study?id=msk_impact_2017

## Abbreviations

AMP: Association for Molecular Pathology
ASCO: American Society of Clinical Oncology
CAP: College of American Pathologists
CGI: Cancer Genome Interpreter
CIViC: Clinical Interpretations of Variants in Cancer
COSMIC: Catalogue of Somatic Mutations in Cancer
MAF: Minor allele frequency
NGS: Next-generation sequencing
NSCLC: Non-small cell lung cancer
VAF: Variant allele frequency/fraction
