# Supplementary material for: Variant Interpretation for Cancer (VIC): a computational tool for assessing clinical impacts of somatic variants
Source: Genome Med. 2019 Aug 23;11:53. doi: 10.1186/s13073-019-0664-4 (PMC6708137; doi:10.1186/s13073-019-0664-4)
Supplement: Supplementary file 3 — More details of the scoring system in VIC. (DOCX 14 kb) [file 13073_2019_664_MOESM3_ESM.docx]

**More details of the scoring system of VIC**

The final decision is made in the function “ClassFy” in the VIC program, the specific algorism is as follows:

1. Strong clinical significance (this is evaluated first to avoid underestimation): “Clinical impacts” is 2, “Mutation type” is 1, “Population database” is 1, “Somatic mutation database” is 2, “Pathway involvement” is 2 and “Germline mutation database” + “Predictive software programs” > 3.
2. Potential clinical significance: “Clinical impacts” > 0, “Mutation type” is 1, “Population database” is 1, “Somatic mutation database” > 0, “Pathway involvement” > 0, “Germline mutation database” + “Predictive software programs” > 1 and at least one of the “Germline mutation database” and “Predictive software programs” not equals to 1.
3. Benign/likely benign: “Clinical impacts” is 0, “Mutation type” is 0, “Population database” is 0, “Germline mutation database” <=1, “Somatic mutation database” is 0, “Predictive software programs” <=1, “Pathway involvement” is 0.
4. Uncertain significance: All the rest scenarios.

The specific scoring methods for the seven criteria (“Clinical impacts”, “Mutation type”, “Population database”, “Germline mutation database”, “Somatic mutation database”, “Predictive software programs” and “Pathway involvement”) are elaborated in the main paper.
